# Supplementary material for: Drug screening on digital microfluidics for cancer precision medicine
Source: Nat Commun. 2024 May 22;15:4363. doi: 10.1038/s41467-024-48616-3 (PMC11111680; doi:10.1038/s41467-024-48616-3)
Supplement: Supplementary file 1 — Supplementary Information [file 41467_2024_48616_MOESM1_ESM.pdf]

**Supporting information for**

# Drug Screening on Digital Microfluidics for Cancer Precision Medicine

Jiao Zhai [1, 2#], Yingying Liu[1, 3#], Weiqing Ji [4#], Xinru Huang[5], Ping Wang[6], Yunyi Li [1], Haoran Li [1,3], Ada Hang-Heng Wong [7], Xiong Zhou [1,8], Ping Chen[9], Lianhong Wang [8], Ning Yang [1, 10], Chi Chen [5], Haitian Chen [5], Pui-In Mak [1,3], Chu-Xia Deng [9], Rui Martins[1,3,11], Mengsu Yang[2], Tsung-Yi Ho [12], Shuhong Yi [5]*, Hailong Yao [4]*, Yanwei Jia [1,3,7,]*.

[1] State Key Laboratory of Analog and Mixed-Signal VLSI, Institute of Microelectronics, University of Macau, Macau SAR, China

[2] Department of Biomedical Sciences, and Tung Biomedical Sciences Centre, City University of Hong Kong, 83 Tat Chee Avenue, Kowloon, Hong Kong SAR, China

[3] Faculty of Science and Technology, University of Macau, Macau SAR, China

[4] School of Computer and Communication Engineering, University of Science and Technology Beijing, Beijing, China

[5] Liver Transplantation Center, The Third Affiliated Hospital, Sun Yat-Sen University, Guangzhou, China

[6] Department of Hepatobiliary Surgery, The First Affiliated Hospital of Guangzhou Medical University, Guangzhou, China

[7] MoE Frontiers Science Center for Precision Oncology, University of Macau, Macau SAR, China

[8] College of electrical and information engineering, Hunan University, Changsha, China

[9] Cancer Center, Faculty of Health Sciences, University of Macau, Macau SAR, China

[10] Department of Electronic Information Engineering, Jiangsu University, Zhenjiang, China

[11] On leave from Instituto Superior Tecnico, Universidade de Lisboa, Lisboa, Portugal

[12] Department of Compute Science and Engineering, The Chinese University of Hong Kong, Hong Kong, China

[#] These authors contributed equally: Jiao Zhai, Yingying Liu, Weiqing Ji

*These authors jointly supervised this work: Shuhong Yi (yishuhong@163.com), Hailong Yao (hailongyao@ustb.edu.cn), Yanwei Jia (yanweijia@um.edu.mo)

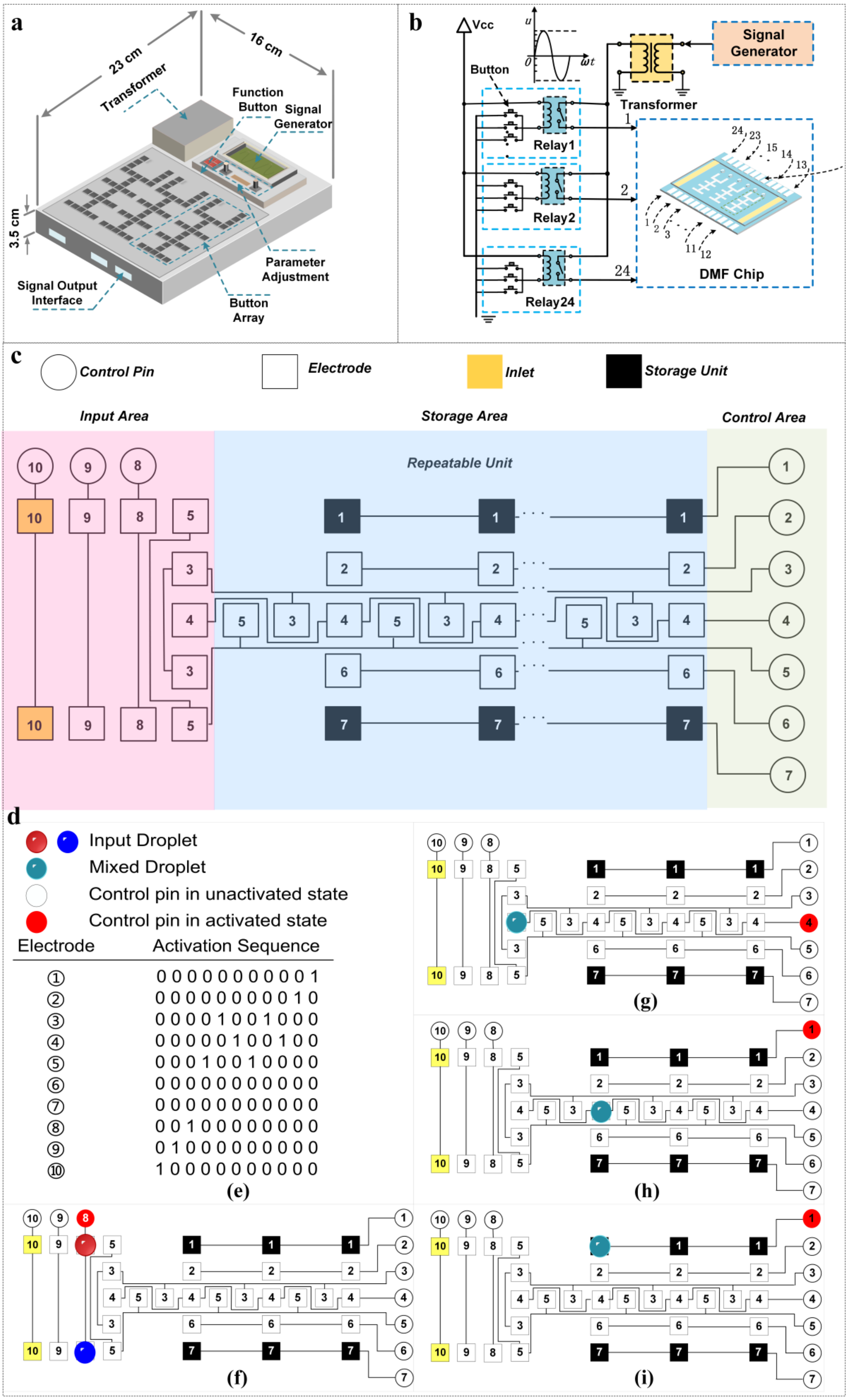


Figure. S1. The scheme and work principle of the portable digital microfluidic system and electrodes

sharing principle on Digital Microfluidic (DMF) chip. a Outlook of the DMF drug screening device. b The principle of the digital microfluidic control system. c, d Smart electrode sharing principle and control algorithm. c Proposed structure of the DMF chip. d Example of moving droplets on a chip with six storage units: (**e**) actuation sequence for electrodes and chip status at (f) t=3, (g) t=6, (h) t=9, and (i) t=11.

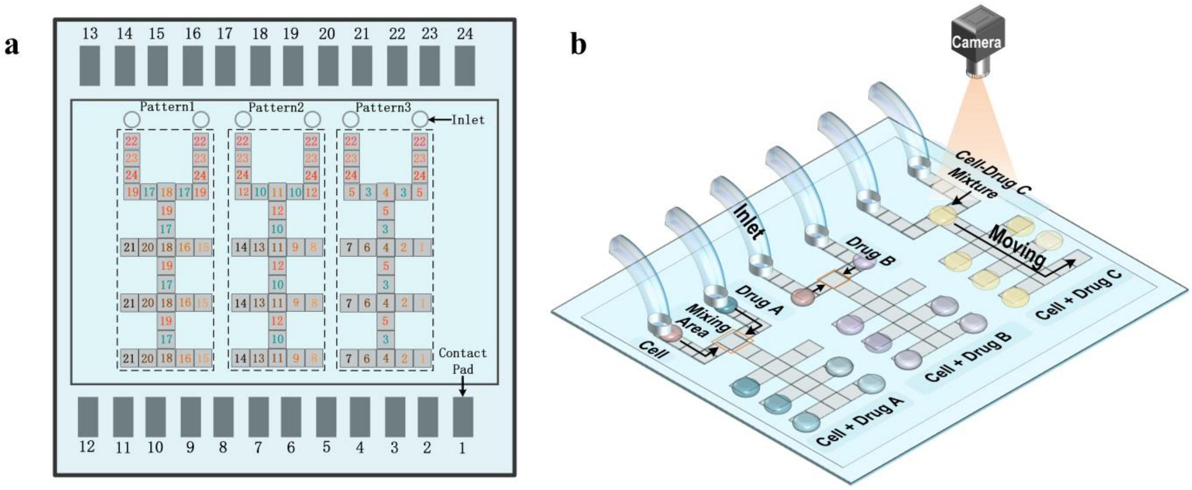


Figure. S2. (a) The Digital Microfluidic (DMF) chip. (b) Scheme of on-chip drug screening.

Two types of biopsy needles were used in this work. Needle #16 (11 mm sample groove) was used in mice samples. Needle #18 (18 mm sample groove) was used in clinical samples. As shown in Fig. S3a, about $1.5\times10^4$ cells were obtained from the xenograft tumor on mice. To quantify the cell numbers obtained from different stages of cancer progress, we obtained samples from three liver cancer patients with two at early stage and one at late stage. As shown in Fig. S3b, the cell numbers do not correlated with the stage of tumors, ranging from $3.8\times10^4$ cells to $6.6\times10^4$ cells, with an average cell number of $5\times10^4$. This is reasonable because the obtained cell number depends on the grove volume on the biopsy needle, not the sample stage.

On DMF chip, we normally used about 300 cells in each droplet for drug screening, but this may not be the lowest number that can provide a reliably drug screening results. To test the limit of cell numbers for valid drug screening, we ran a serial dilution of cell numbers from 100 to 100,000 in the presence of drug in a 96-well microplate or on-chip. MDA-MB-231 was used as the cell model and EP as the drug model. AlamarBlue® assay was used for the cell toxicity quantification on 96-well plates. Briefly, a serious of cell numbers ($1.0\times10^5$ cells, $1.0\times10^4$ cells, $5.0\times10^3$ cells, $10^3$ cells, $5.0\times10^2$ cells, $1.0\times10^2$ cells, the total volume was 100

μl) per well were seeded in a 96-well plate in the DMEM cell culture medium. They were then treated with various concentrations of EP (with 0.1% (v/v) dimethyl sulfoxide (DMSO) treatment as a negative control and a cell culture medium without cells as a blank control) for 24 hours. Then, 10 μl of alamarBlue solution was added to each well and incubated for 2 h. All experiments were performed in triplicate. Finally, 585 nm emission was measured by a microplate reader (with the fluorescence excitation wavelength of 555 nm). The emission values were reduced by the blank and normalized to the control wells. Graphs were plotted as the drug concentration versus the percentage of viable cells. As shown in Fig. S3c, the cell viability decreased with increasing the drug concentration in each group of different cell numbers. The $IC_{50}$ values were comparable to each other when the cell number was more than 1000. When the cell number was lowered to 500 cells per sample, the deviation was obviously enlarged and the $IC_{50}$ value increased a lot. The reliable cell required for 96-well plate is about 1000 cells. Compared to the 100 μL solution volume in 96-well plate, 384-well plate takes 50 μL solutions per sample. The biopsied cells may be enough for a screening of one drug with 6 conditions in a 384-well plate. One drug screening would not provide useful information for precision medicine.

We further tested the drug screening with 100 cells in a droplet on DMF chip. No higher cell numbers were tested due to the droplet accommodation. As shown in Fig S3d, the cell viability curve was similar as that with 300 cells.

As shown in Fig. S3c and Fig. S3d, there are subtle different responses for on-chip and off-chip to the same increased concentration of drugs, which may be attributed to the different platforms. However, the $IC_{50}$ for on-chip and off-chip were both around 25 μM under optimal conditions, validating the on-chip drug screening indication.

We expect the DMF chip can handle as least as 100 cells per condition. Our data confirmed that 100 cells can give a similar result as 300 cells.

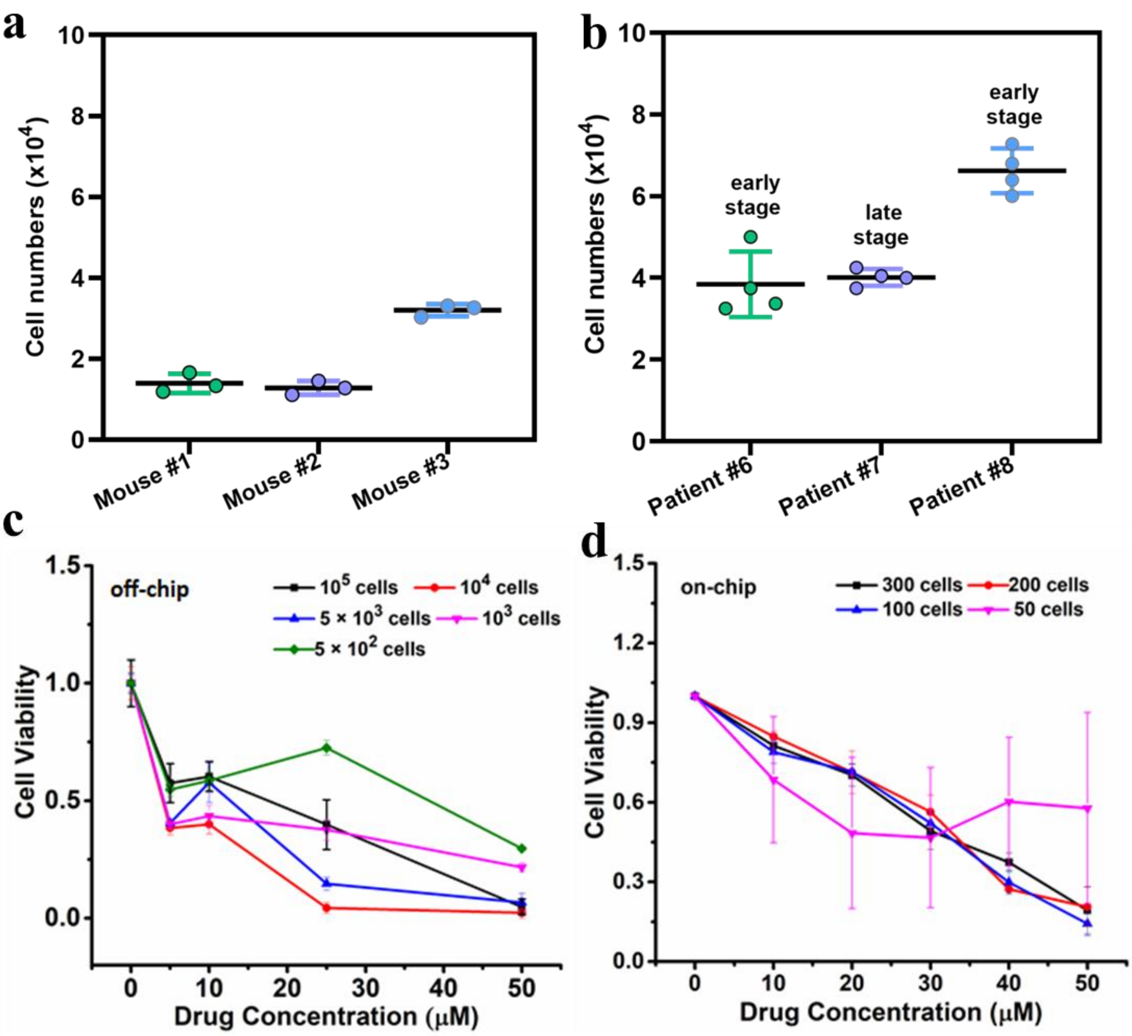


Figure. S3. Cell count of each biopsies from mice (a) (n=3 independent experiments) and human patients (b) (n=4 independent experiments). (c) Cell Viability results of a serious of MDA-MB-231 cell numbers ($1.0\times10^5$ cells, $1.0\times10^4$ cells, $5.0\times10^3$ cells, $10^3$ cells, $5.0\times10^2$ cells) after drug epirubicin hydrochloride (EP) (0 μM, 5 μM, 10 μM, 25 μM, 50 μM) treatment based on well-plates method, n=3 independent experiments. (d) Cell Viability results of a serious of MDA-MB-231 cell numbers (300 cells, 200 cells, 100 cells, 50 cells) after drug EP (0 μM, 10 μM, 20 μM, 30 μM, 40 μM, 50 μM) treatment based on chip method, n=3 independent experiments. Source data are provided as a Source Data file.

Fig. S4 showed the detailed drug screening results of Dox alone, Cur alone, and the combination of 10 μM Dox and various concentration of Cur. As can be seen, Dox alone worked better than Cur alone in most cases with lower cell viabilities. The combination of two drugs always showed better effect than single drug, with lower cell viabilities.

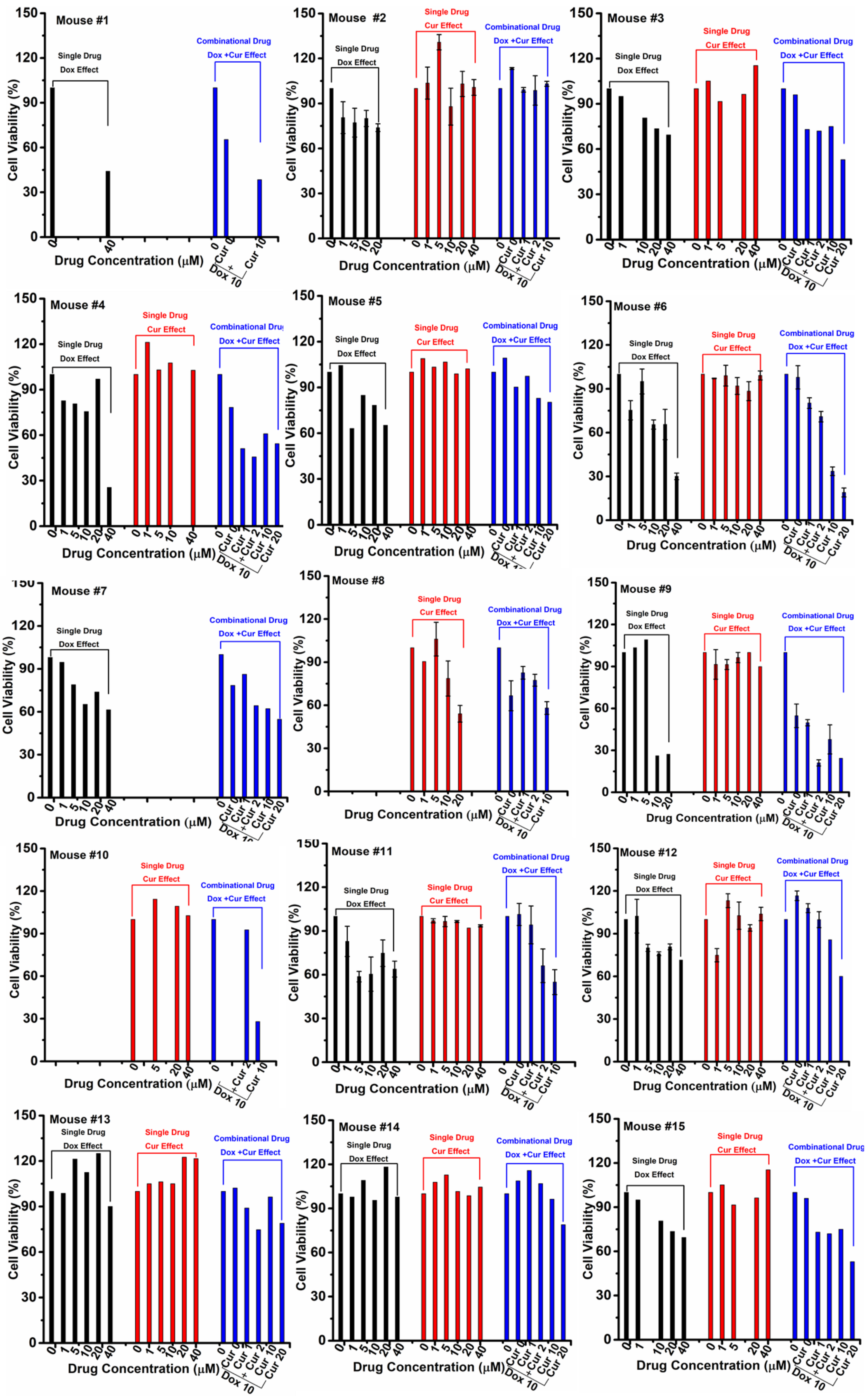


Figure. S4 On-chip single drug and combinational drug screening results of biospy samples from 15 individual mice. The drugs are Doxorubicin (Dox), Curcumol and Doxorubicin (Dox) plus Curcumol. For

mouse #1, 3, 4, 5, 7, 10, 13-15, n=1; For mouse #2, 6, 8, 9, 11,12, n=2. Source data are provided as a Source Data file.

The gene mutation for the patients was detected by Whole-exome sequencing (WES). The exactly mutation for Patient #1 and Patient #5 was shown in following Fig. S5.

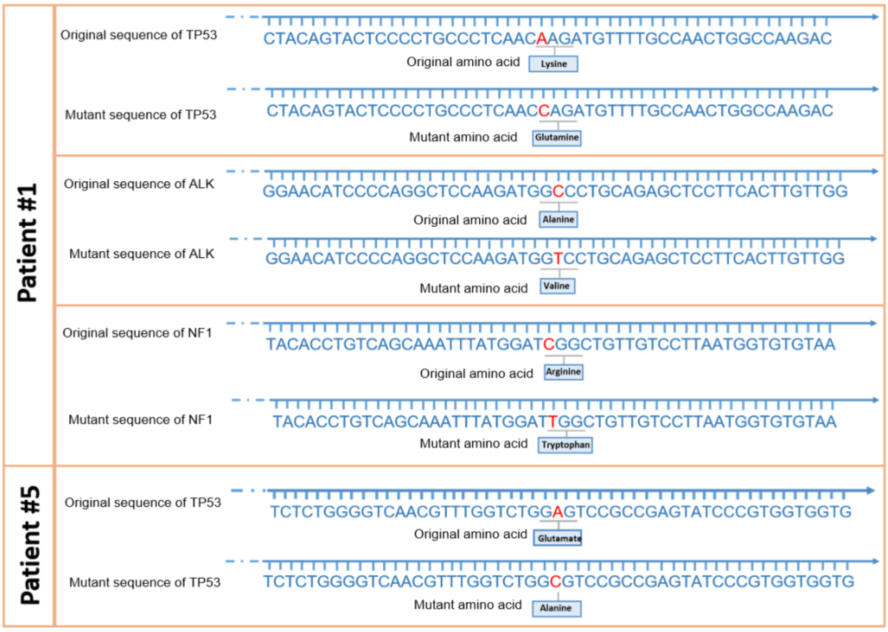


Figure. S5. The exact mutation for the patients.

Histological image results for patients #1 to #5 are shown in the following Fig. S6. The tumor resection was marked with yellow circle. The CT images of patients #1 #3, #4, #5 (Patient #2 didn't do CT image after surgey) are shown in Fig. S7. It can be seen that the tumors have been clearly resected after surgery.

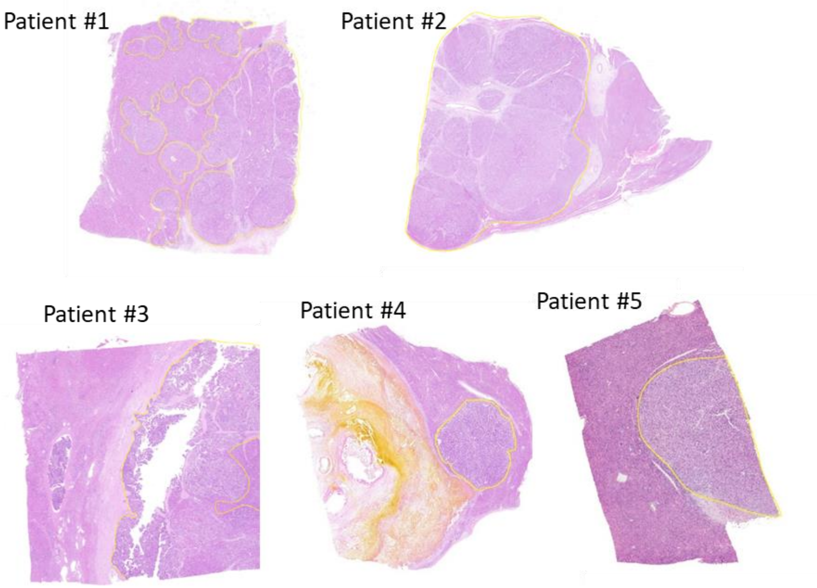


Figure. S6. Histological image results for patients #1 to #5. Cancer cells are highlighted with yellow area.

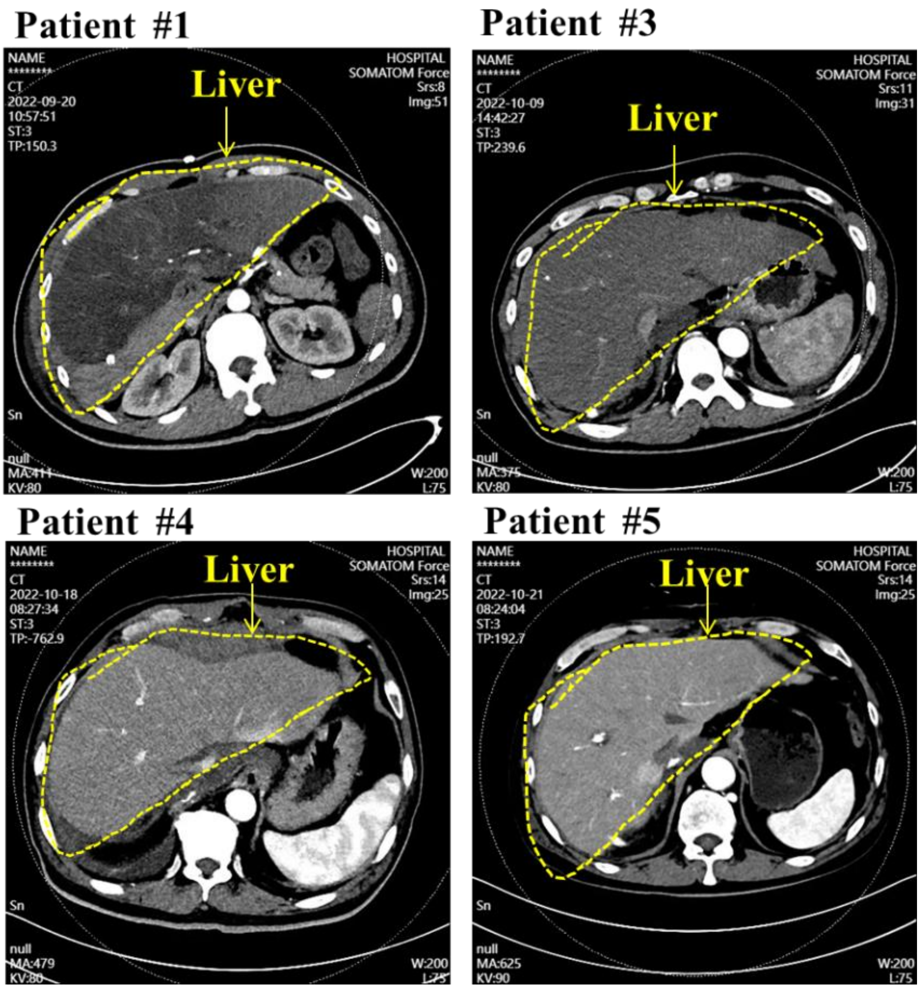


Figure. S7. Computerized tomography (CT) images of patient#1, #3, #4, #5. The yellow circle represents patient liver.

To avoid hypoxia, the requirements are to maintain sufficient oxygen supply and eliminate factors that consume oxygen. Primary cells can be exposed to hypoxic conditions for a certain period of time before damage occurs, but this varies depending on cell type and experimental conditions. Generally speaking, the longer the exposure time, the greater the risk of damage to the cells.

To figure out that how long can primary tumor cells be exposed to hypoxic conditions before damage sets in, we designed an experiment. A relatively large tumor tissue with good initial activity was chosen and put in a 50 ml tube with no air in the tube to create hypoxic conditions (Fig. S8a). Then we cut off a piece of tumor tissues at different time points (0 h, 6 h, 12 h), dissociated them into single cells, and checked the cell activity. As shown in Fig. S8b, the cell viability remained at about 70% when the primary liver cancer cells were kept in a relatively hypoxic environment for 6 hours. There was no big difference between 0 hour and 6 hours. However, cell viability decreased sharply after 6 hours and was less than 10% at 12 hours (Fig. S8c). This indicates that damage occurs after 6 hours of hypoxia condition.

For the highest cell viability in drug screening, the most fresh cells the best results. The longest period for the tumor to be kept without any treatment was 6 hours.

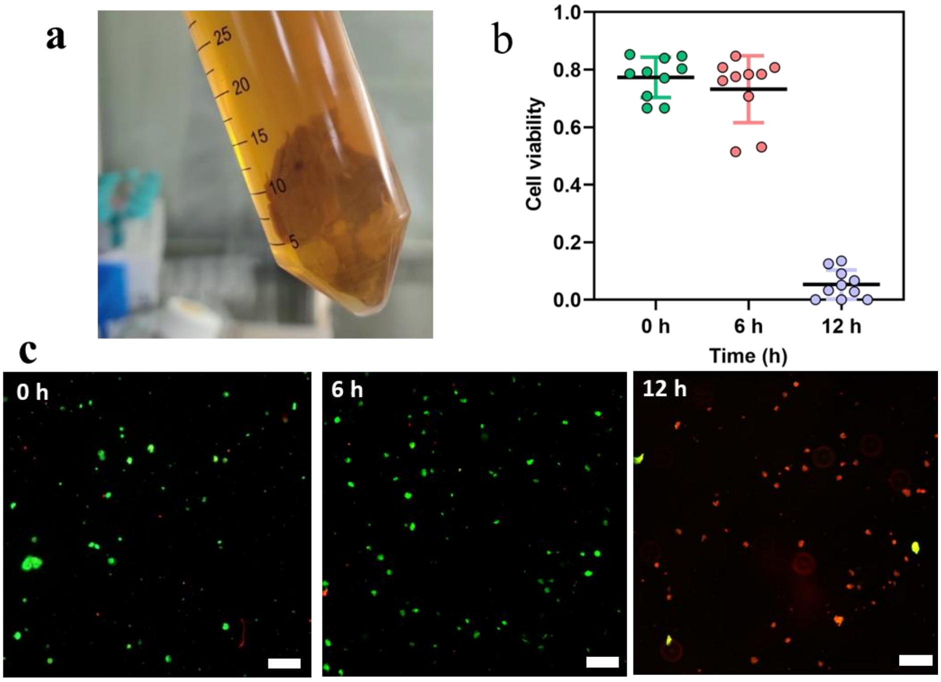


Figure. S8. Cell viability measurement of the primary tumor cells exposed to hypoxic conditions. (a). Picture of tumor tissue from patient #9 in hypoxia condition. (b). Corresponding chart showed cell viability from primary liver cancer samples after exposed to hypoxia condition for 0-12 h. n=10 independent experiments. (c). Fluorescent image results of dissociated cells from primary liver cells after exposed to

hypoxia condition for 0-12 h. Green represents living cells and red represents dead cells. Scale bars are 100 μm. Source data are provided as a Source Data file.

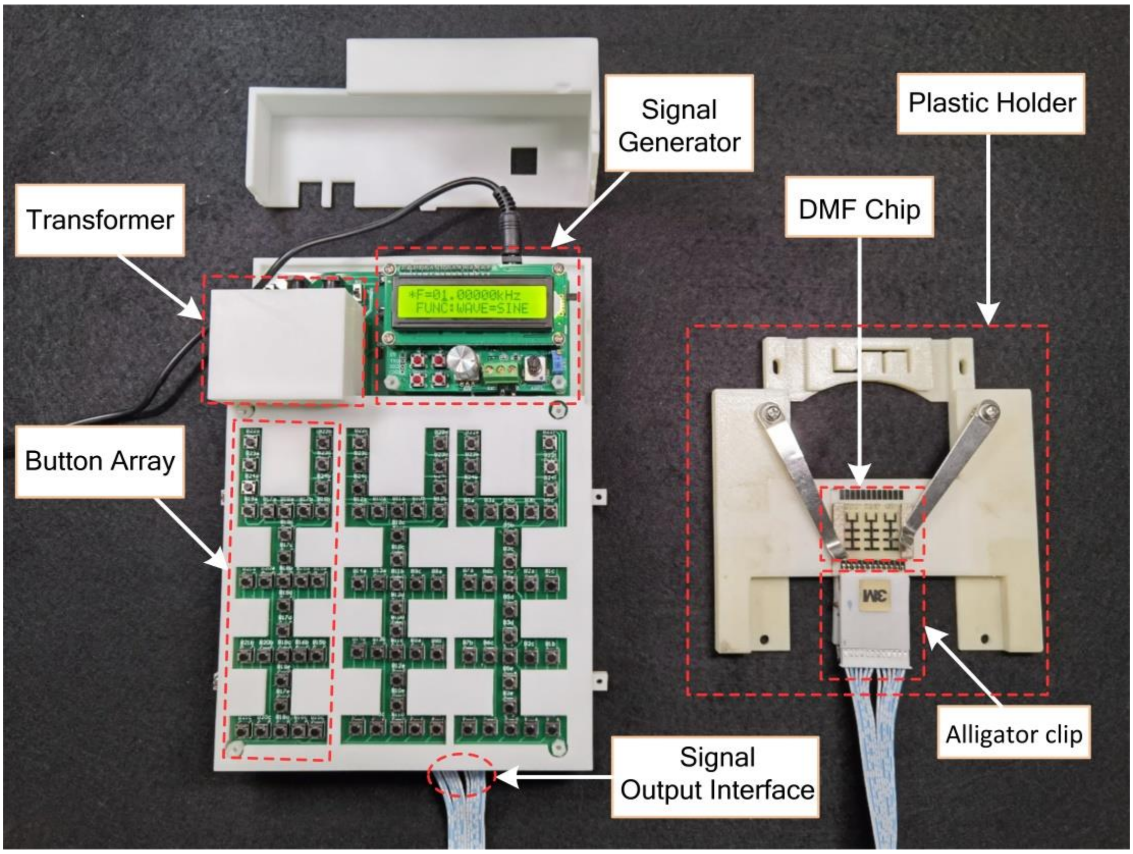


Figure. S9 The image picture of portable digital microfluidic system and its connection with digital microfluidic chip to provide power supply for droplet actuation. The digital microfluidic system mainly includes four parts: transformer, signal generator, button array and signal output interface. The portable digital microfluidic system is connected with Digital Microfluidic (DMF) chip via alligator clip.

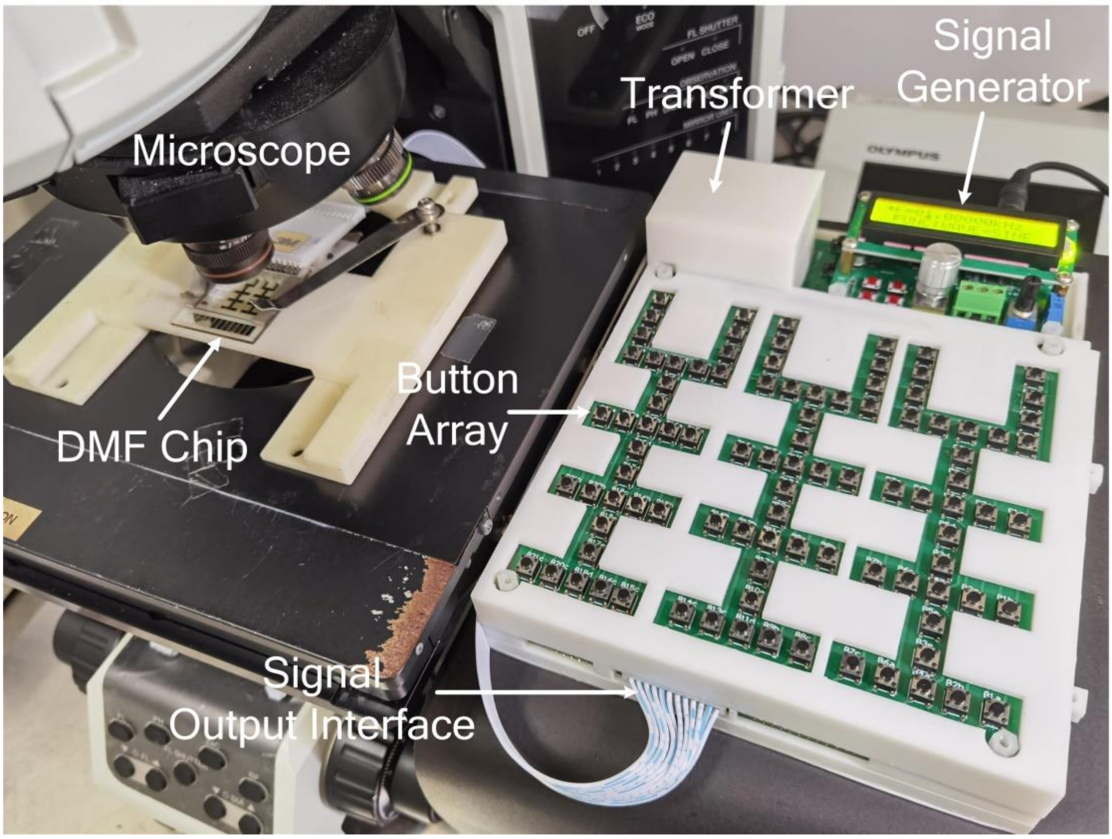


Figure. S10. The image picture for droplet operation on Digital Microfluidic (DMF) chip. Microscope was used to observe the droplet operation on DMF chip charged by the portable digital microfluidic system. The digital microfluidic system mainly includes four parts: transformer, signal generator, button array and signal output interface. The portable digital microfluidic system is connected with DMF chip via alligator clip.

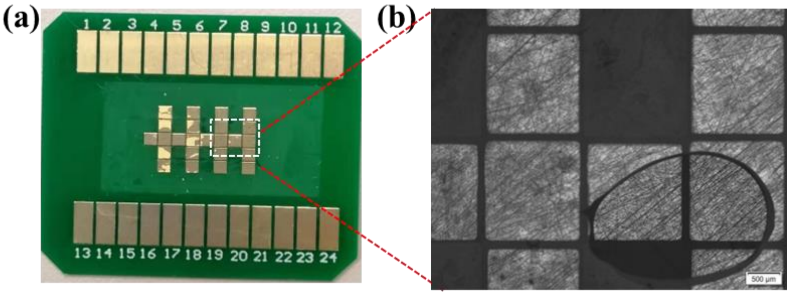


Figure. S11. The image result of printed circuit board (PCB) substrate observed under natural light (a) and the enlarged substrate area observed under microscopy (b).

To characterize what was obtained from the biopsies of mice, we did HE staining analysis. The paraffin-embedded tumor slides with 5 μm thickness were analyzed by immunohistochemical method. The tissues were deparaffinized in xylene, rehydrated with a graded series of ethanol, and rinsed in water, following by staining with Hematoxylin stain

(Harris) for 3-5 min, and rinsed in water for 1-2 min, differentiation with 0.8% ~ 1% hydrochloric acid alcohol and rinsing with water. Subsequently, the tissues were stained with eosin stain (alcohol soluble) for 1-2 sec, and dehydrated with 95% ethanol and anhydrous ethanol for 1-2 minutes. Finally, the tissues were trans parented with xylene, sealed, and observed with microscopy. The results in Fig. S12 suggested almost all of the cells were tumor cells, with the evidence of the similar morphology of cells in the slides and big nuclear to cytoplasmic ratio of the cells.

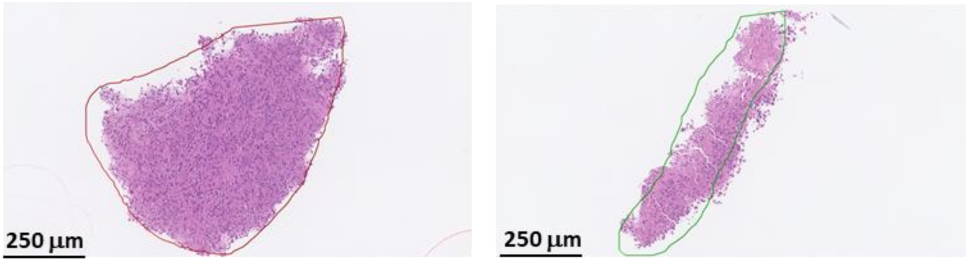


Figure. S12. HE staining results of the biopsy samples from two mice, n=2 independent experiments.
