# Supplementary material for: Drug screening on digital microfluidics for cancer precision medicine
Source: Nat Commun. 2024 May 22;15:4363. doi: 10.1038/s41467-024-48616-3 (PMC11111680; doi:10.1038/s41467-024-48616-3)
Supplement: Supplementary file 3 — Description of Additional Supplementary Files [file 41467_2024_48616_MOESM3_ESM.pdf]

<u>**Description of Additional Supplementary Files**</u>

Supplementary Movie 1: Demonstration for one among three parallel drugs screening on the chip.

Supplementary Movie 2: Droplet movement based on electrode-sharing principle.

Supplementary Movie 3: Droplet actuation on DMF chip.

Supplementary Movie 4: Movement of a drop with cells. Supplementary Movie 5: Droplet collection from the culture spot.

Supplementary data 1: The original sequence file of Patient #1.

Supplementary data 2: The original sequence file of Patient #3.

Supplementary data 3: The original sequence file of Patient #5.
