# Supplementary material for: Drug screening on digital microfluidics for cancer precision medicine
Source: Nat Commun. 2024 May 22;15:4363. doi: 10.1038/s41467-024-48616-3 (PMC11111680; doi:10.1038/s41467-024-48616-3)
Supplement: Supplementary file 4 — Supplementary Data 1 [file 41467_2024_48616_MOESM4_ESM.docx]

| **#Gene** | **cHGVS** | **pHGVS** | up100 (ref/alt) down100 |
| --- | --- | --- | --- |
| TP53* | c.394A>C | p.K132Q | TGCTTGTAGATGGCCATGGCGCGGACGCGGGTGCCGGGCGGGGGTGTGGAATCAACCCACAGCTGCACAGGGCAGGTCTTGGCCAGTTGGCAAAACATCT(T/G)GTTGAGGGCAGGGGAGTACTGTAGGAAGAGGAAGGAGACAGAGTTGAAAGTCAGGGCACAAGTGAACAGATAAAGCAACTGGAAGACGGCAGCAAAGAAA  Reverse complement：  TTTCTTTGCTGCCGTCTTCCAGTTGCTTTATCTGTTCACTTGTGCCCTGACTTTCAACTCTGTCTCCTTCCTCTTCCTACAGTACTCCCCTGCCCTCAAC(C/A)AGATGTTTTGCCAACTGGCCAAGACCTGCCCTGTGCAGCTGTGGGTTGATTCCACACCCCCGCCCGGCACCCGCGTCCGCGCCATGGCCATCTACAAGCA |
| ALK* | c.1307C>T | p.A436V | CTGGCTCTCATCTTCTCCCTGGGCACAGTCCTGGTGGAAGTCACAGGCCTGCCCAAGCTGGAGGACTGTCCCATTCCAACAAGTGAAGGAGCTCTGCAGG(G/A)CCATCTTGGAGCCTGGGGATGTTCCTGGAGAGCACACAGACACACAACCATGGTAAGTTTGCATGGCCCCAGGCAGCAGCTGGCCTGATGGGTTGGTCCC  Reverse complement：  GGGACCAACCCATCAGGCCAGCTGCTGCCTGGGGCCATGCAAACTTACCATGGTTGTGTGTCTGTGTGCTCTCCAGGAACATCCCCAGGCTCCAAGATGG(T/C)CCTGCAGAGCTCCTTCACTTGTTGGAATGGGACAGTCCTCCAGCTTGGGCAGGCCTGTGACTTCCACCAGGACTGTGCCCAGGGAGAAGATGAGAGCCAG |
| NF1* | c.2689C>T | p.R897W | TAGCCCACCCATGGGTCCAGTCAGTGAACGTAAGGGTTCTATGATTTCAGTGATGTCTTCAGAGGGAAACGCAGATACACCTGTCAGCAAATTTATGGAT(C/T)GGCTGTTGTCCTTAATGGTGTGTAACCATGAGAAAGTGGGACTTCAAATACGGACCAATGTTAAGGATCTGGTGGGTCTAGAATTGAGTCCTGCTCTGTA |
| HRAS | c.279C>T | p.I93= | CAGAGAGGACAGGAGGCCCCTGCCTGGACGCAGCCGGCCTGGCCCCACCTGTGCGGCGTGGGCTCCCGGGCCAGCCTCACGGGGTTCACCTGTACTGGTG(G/A)ATGTCCTCAAAAGACTTGGTGTTGTTGATGGCAAACACACACAGGAAGCCCTCCCCGGTGCGCATGTACTGGTCCCGCATGGCGCTGTACTCCTCCTGGC |
| PTPRD | c.4875G>C | p.L1625F | AATAACCACATCACATCCAATACCATACCTTAAATTCGAGCTCCATTCCTGTGACATTCTCTCCCGTTTCTATTTGTGTCAGCTTCTGAATGTAGGCATA(C/G)AAGTTTCTAGCTGGCACTTCGGTATTTCCACAAGTCACTGCTTCTAACAGTGCATCATGGATAAAGATGTATTGGTCTTCTGTTTGAACCATATAGTTCC |
| ZNF804A | c.2037T>A | p.T679= | CAGAGCAATTATTAGACTCACATCAGTTACTTGATAAAAGGCCCAAATCAGAATCCATATCCTTAAGTGACAATGAAGAAATGTGTAAAACATGGAATAC(T/A)GAATACAACACTTATGATACTATCAGTTCTAAAAACCACTGTAAAAAGAACACAATACTTTTAAATGGACAATCAAATGCAACAATGATACATTCTGGGA |
| SLC34A2 | c.1372A>G | p.T458A | CACCACTGCCATTTCCTGTCATCCCATGGGGCTGATATGTTTGTGTTTTGTGTTTCCCCCAGGAATCGGCGTGATAACCATTGAGAGGGCTTATCCACTC(A/G)CGCTGGGCTCCAACATCGGCACCACCACCACCGCCATCCTGGCCGCCTTAGCCAGCCCTGGCAATGCATTGAGGAGTTCACTCCAGGTCAGGACTTGGGG |
| ALK | c.4026C>G | p.V1342= | TGAAAAGAAAAACTGCTTAGTAACTAGCAGAAGTGTTCCTAAAAGAGTCATACACAGGCCCAGGGCAGTTCTTGGGTGGGTCCATCCGGCCTCCACTGGT(G/C)ACAAACTCCAGAACTTCCTGGTTGCTTTTGCTGGGGTATGGCATATATCCAAGAGAAAAGATTTCCCATAGCAGCACTCCAAAGGACCTGGGCATGGGAC |
| ZNF804A | c.3084T>G | p.A1028= | ATTCAGGAATCCTTAACACACAACCACCATTACCATTCAAAGAAGCACATGTCAGTGGTCATACTTTTGTAACAGCTGAGCAAATCCTGGCTCCATTAGC(T/G)TTACCAGAGCAAGCATTATTGATCCCACTAGAAAACCATGACAAATTCAAAAATGTACCATGTGAGGTCTACCAGCACATTCTGCAGCCAAACATGCTGG |
| ABL2 | c.1262_1267delATGTGGinsCTAATGTGA | p.H421_V423delinsPNVI | CTCTCTGGTGCTGTCCACTTAATAGGAAATTTGGCTCCAGCATGAGCAGTATAAGTGTCTCCAGTCATCAATCTACTTAAGCCAAAGTCAGCCACTTTTA(CCACAT/TCACATTAG)GGTTTTCTCCCACTAGGCAGTTACGAGCTGCAAGATCTCTGTGGGAAAGAGAACCCTAATGTGATTCCATTCAGATGGTGAGGAAAATGGTCATGAACAC |
| NOTCH1 | c.6751G>A | p.A2251T | GTGCTGGTGCCAGAGGCCACAGGCAGGTGGGAGAGACGAGGTGGGCCAGTCTCAAAGGCCAGCCGGCCGCCCCCACCCAGCGCCGCCATCTCGGGCTTGG(C/T)CGCCACGTTCAGGTGCCCGATGCCCAGGTGGGTGTCGGGCATCCCAGGCAGGTGGTTGAGGGGCACGGACGGAGACTGCTGGAACGGGGAGGGCAGCAGT |
| APC | c.302G>A | p.G101E | TTGTTTCTATTTTATTTAGAGCTTAACTTAGATAGCAGTAATTTCCCTGGAGTAAAACTGCGGTCAAAAATGTCCCTCCGTTCTTATGGAAGCCGGGAAG(G/A)ATCTGTATCAAGCCGTTCTGGAGAGTGCAGTCCTGTTCCTATGGGTTCATTTCCAAGAAGAGGGTTTGTAAATGGAAGCAGAGAAAGTACTGGATATTTA |
