# Supplementary material for: Drug screening on digital microfluidics for cancer precision medicine
Source: Nat Commun. 2024 May 22;15:4363. doi: 10.1038/s41467-024-48616-3 (PMC11111680; doi:10.1038/s41467-024-48616-3)
Supplement: Supplementary file 5 — Supplementary Data 2 [file 41467_2024_48616_MOESM5_ESM.docx]

| **Gene** | **cHGVS** | **pHGVS** | **up100 (ref/alt) down100** |
| --- | --- | --- | --- |
| OR4C46 | c.920G>T | p.G307V | ACTATGATAACTCCTATGTTAAACCCCTTAATCTATACCTTGAAGAATGCCCAGATGAAAAATGCCATCAGGAAATTGTGTAGTAGAAAGGACATTTCAG (G/T) TGACAAATAAATGTAACTAGAGCTCAACATTGATTCAATTTAGtcatgtccttttagggacatggatgaagctggaaatcatcattctcagctaactatc |
| CYP4B1 | c.518G>A | p.R173Q | GGAGGGCAGCTTGGGGCATCCAGCCCAACTAACCCCTGCATCGCCTCCTACACATTGCCTCCTATCCCTGGACTCCAGGACAAGTGGGAAGAGAAAGCTC (G/A) GGAGGGTAAGTCCTTTGACATCTTCTGCGATGTGGGTCACATGGCGCTGAACACACTCATGAAGTGCACCTTTGGAAGAGGAGACACCGGCCTGGGCCAC |
| OR2L8 | c.820G>T | p.A274S | CAGCACCCACCTCACTGTAGTAACTTTCTACTATGCACCTTTTGTCTACACTTATCTACGTCCAAGATCCCTGCGATCTCCAACAGAGGACAAGGTTCTG (G/T) CTGTCTTCTACACCATCCTCACCCCAATGCTCAACCCCATCATCTATAGCCTGAGGAACAAGGAGGTGATGGGGGCCCTGACACGAGTGAGTCAGAGAAT |
| POM121L12 | c.146G>A | p.R49H | TGGAAGGCGGGAGAACCCCTGCTGCAAGGCCCCGACGCCCTGGCGGCTCCCATGAGCAGGTCACCCAGCACGCCCCAGACCACGCCATCTCCCCAGGGTC (G/A) CCAGAGTCCCTGGCCCCTGAGGTCCCTGACTCAGAGCCATATTCAGTACTTCCAGTGGGGGCGCCCGGTGCCCAGCACCCACCTCATCGAGGTGCGGCCC |
| EPHA3 | c.1774G>A | p.G592S | TTTTTAAACCAATAATGCCATAAAATTTGATCTATAATTGTTTGTACAAATCTAGCTACAATTGCGCCTTTCTTTCTTTCCTCAAACAGTAAAACTTCCA (G/A) GTCTCAGGACTTATGTTGACCCACATACATATGAAGACCCTACCCAAGCTGTTCATGAGTTTGCCAAGGAATTGGATGCCACCAACATATCCATTGATAA |
| OR2T34 | c.748A>G | p.M250V | TAAAAGGCAGACACCATCATGTCCTGCTCAGCTGTGTGGTAGGAACTCCGGAGCATGTAGGTGTAGAAGGAAGCACCGAAGAGCAGCAGCACTATGATCA (T/C) GTGGGAGGAGCAGGTGGCCAAGGCCTTCCTGCGGCCGGCGGCAGAATTCATCCTGTGGATGAGATGCAGGATGAGGGTGTATGAGCTGGAGATGACCATG |
| OCA2 | c.1517C>T | p.A506V | CTTGTTATAAAGCTTTCTGTTCCAGTAAAGGAGTCTGAGGAGCGGAAAGCAGACCAGGAGAACAAGGCAAATCCCAATGAACATGTGTGCAGTGAATCCG (G/A) CAAAGTCCAGGCCCTGGAAATAAACAAGGGGAAATGAAATGGCAGCccaggcatggtggctcacgcctgtaatcccagcactttccgaggtgggtggatc |

Copy number vibration ：

| **Gene** | **Copy Number** | **Remarks** |
| --- | --- | --- |
| MET* | 4.05 | No nucleotide mutation sequence |
