# Supplementary material for: Drug screening on digital microfluidics for cancer precision medicine
Source: Nat Commun. 2024 May 22;15:4363. doi: 10.1038/s41467-024-48616-3 (PMC11111680; doi:10.1038/s41467-024-48616-3)
Supplement: Supplementary file 6 — Supplementary Data 3 [file 41467_2024_48616_MOESM6_ESM.docx]

| **#Gene** | **cHGVS** | **pHGVS** | **up100 (ref/alt) down100** |
| --- | --- | --- | --- |
| TP53* | c.671A>C | p.E224A | AAATAAGCAGCAGGAGAAAGCCCCCCTACTGCTCACCTGGAGGGCCACTGACAACCACCCTTAACCCCTCCTCCCAGAGACCCCAGTTGCAAACCAGACC(T/G)CAGGCGGCTCATAGGGCACCACCACACTATGTCGAAAAGTGTTTCTGTCATCCAAATACTCCACACGCAAATTTCCTTCCACTCGGATAAGATGCTGAGG  complementary sequences：  TCTCTGGGGTCAACGTTTGGTCTGG(A)GTCCGCCGAGTATCCCGTGGTGGTG |
| NOTCH1 | c.6714C>T | p.P2238= | GAGGTGGGCCAGTCTCAAAGGCCAGCCGGCCGCCCCCACCCAGCGCCGCCATCTCGGGCTTGGCCGCCACGTTCAGGTGCCCGATGCCCAGGTGGGTGTC(G/A)GGCATCCCAGGCAGGTGGTTGAGGGGCACGGACGGAGACTGCTGGAACGGGGAGGGCAGCAGTGGCGGCGAGGCCACGTCTGACAGGTAGCCATGGGGTG |
| OR6F1 | c.542T>C | p.I181T | GGTGATGAGGCATGAACTCAGGATAACCACAACAGCAATCACAAAGGCCACAAGCTCTACTGCCTGTGTGTTGGTGCAGGCCAGGGCAATCCAGGGTGCA(A/G)TGTCACAGAAGAAGTGGTTGATGGCACGGGGGCCACAGAAGGACAGGCCACTGATGAGGGCTGTGGGCACTGCAATGGCCACGAAACCACACACCCAGGA |
| ATM | c.2075G>A | p.R692H | AAGATGGACTTTTTAACCATTGTGAGAGAATGTGGTATAGAAAAGCACCAGTCCAGTATTGGCTTCTCTGTCCACCAGAATCTCAAGGAATCACTGGATC(G/A)CTGTCTTCTGGGATTATCAGAACAGCTTCTGAATAATTACTCATCTGAGGTGAGATTTTTTAAAAAAAGAACTAAGCTTATATATGATTCAACTTTGGTA |
| APC | c.8080dupA | p.I2694Nfs*2 | AATTGAGGACTGTCCCATTAACAATCCTAGATCTGGAAGATCTCCCACAGGTAATACTCCCCCGGTGATTGACAGTGTTTCAGAAAAGGCAAATCCAAAC(A/AA)TTAAAGATTCAAAAGATAATCAGGCAAAACAAAATGTGGGTAATGGCAGTGTTCCCATGCGTACCGTGGGTTTGGAAAATCGCCTGAACTCCTTTATTCA |
