# Supplementary material for: Drug screening on digital microfluidics for cancer precision medicine
Source: Nat Commun. 2024 May 22;15:4363. doi: 10.1038/s41467-024-48616-3 (PMC11111680; doi:10.1038/s41467-024-48616-3)
Supplement: Supplementary file 12 — Reporting Summary [file 41467_2024_48616_MOESM12_ESM.pdf]

nature portfolio

Corresponding author(s): Yanwei Jia

Last updated by author(s): April 18, 2024

Reporting Summary

Nature Portfolio wishes to improve the reproducibility of the work that we publish. This form provides structure for consistency and transparency in reporting. For further information on Nature Portfolio policies, see our Editorial Policies and the Editorial Policy Checklist.

Statistics

For all statistical analyses, confirm that the following items are present in the figure legend, table legend, main text, or Methods section.

n/a	Confirmed	
☐	☒	The exact sample size (*n*) for each experimental group/condition, given as a discrete number and unit of measurement
☐	☒	A statement on whether measurements were taken from distinct samples or whether the same sample was measured repeatedly
☐	☒	The statistical test(s) used AND whether they are one- or two-sided<br>*Only common tests should be described solely by name; describe more complex techniques in the Methods section.*
☐	☒	A description of all covariates tested
☒	☐	A description of any assumptions or corrections, such as tests of normality and adjustment for multiple comparisons
☐	☒	A full description of the statistical parameters including central tendency (e.g. means) or other basic estimates (e.g. regression coefficient) AND variation (e.g. standard deviation) or associated estimates of uncertainty (e.g. confidence intervals)
☐	☒	For null hypothesis testing, the test statistic (e.g. *F*, *t*, *r*) with confidence intervals, effect sizes, degrees of freedom and *P* value noted<br>*Give P values as exact values whenever suitable.*
☒	☐	For Bayesian analysis, information on the choice of priors and Markov chain Monte Carlo settings
☒	☐	For hierarchical and complex designs, identification of the appropriate level for tests and full reporting of outcomes
☐	☒	Estimates of effect sizes (e.g. Cohen's *d*, Pearson's *r*), indicating how they were calculated

Our web collection on statistics for biologists contains articles on many of the points above.

Software and code

Policy information about availability of computer code

Data collection	The microscopy image data was collected using NIS-Elements on a Nikon microscope and Cellsens Dimension on an Olympus microscope.
Data analysis	We use Origin 8.0, ImageJ, Prism, NIS-Elements, and Cellsens Dimension for data and image analysis.

For manuscripts utilizing custom algorithms or software that are central to the research but not yet described in published literature, software must be made available to editors and reviewers. We strongly encourage code deposition in a community repository (e.g. GitHub). See the Nature Portfolio guidelines for submitting code & software for further information.

Data

Policy information about availability of data

All manuscripts must include a data availability statement. This statement should provide the following information, where applicable:

- Accession codes, unique identifiers, or web links for publicly available datasets
- A description of any restrictions on data availability
- For clinical datasets or third party data, please ensure that the statement adheres to our policy

All the data supporting the findings of this study can be found within the supplementary files, source datas. The original image data are available upon request. A reporting summary for this article is available as a Supplementary Information file.

Research involving human participants, their data, or biological material

Policy information about studies with human participants or human data. See also policy information about sex, gender (identity/presentation), and sexual orientation and race, ethnicity and racism.

Reporting on sex and gender	Findings apply to both sex and genders. Consent has been obtained sharing individual level data. In this study, totally 5 participants were involved.
Reporting on race, ethnicity, or other socially relevant groupings	Race, ethnicity, or other social relevant factors are not considered in this study.
Population characteristics	See above
Recruitment	Patient are selected based on the criteria that they are over 18 years old and should have a tumor that can be resected or be biopsied to provide primary tumor cells for on-chip drug screening.
Ethics oversight	University of Macau

Note that full information on the approval of the study protocol must also be provided in the manuscript.

Field-specific reporting

Please select the one below that is the best fit for your research. If you are not sure, read the appropriate sections before making your selection.

☒ Life sciences ☐ Behavioural & social sciences ☐ Ecological, evolutionary & environmental sciences

For a reference copy of the document with all sections, see nature.com/documents/nr-reporting-summary-flat.pdf

Life sciences study design

All studies must disclose on these points even when the disclosure is negative.

Sample size	For the nude mice treatment research, at least five mice were needed in the experimental group. We used seven mice in each treatment group for single drug screening research part and five mice in each treatment group for the combinational drug screening research part.
Data exclusions	The mice with tumors larger or smaller than the majority were discarded in the experiment. The mice with tumors larger than 300 mm3(454.9496 mm3, 365.3926 mm3, 342.4013 mm3) or smaller than 100 mm3 (40.768 mm3, 89.105 mm3, 85.0023 mm3, 97.3814 mm3, 75.504 mm3, 49.3293 mm3,) were not used for the experiments.
Replication	All experiments were conducted at least three times independently, and similar results were adopted for further analysis to guarantee reproducibility.
Randomization	The nude mice allocation to each treatment group is randomization.
Blinding	Investigators were blinded to the in vivo drug treatment experiments.

Reporting for specific materials, systems and methods

We require information from authors about some types of materials, experimental systems and methods used in many studies. Here, indicate whether each material, system or method listed is relevant to your study. If you are not sure if a list item applies to your research, read the appropriate section before selecting a response.

Materials & experimental systems

n/a	Involved in the study	
☐	☒	Antibodies
☐	☒	Eukaryotic cell lines
☒	☐	Palaeontology and archaeology
☐	☒	Animals and other organisms
☐	☒	Clinical data
☒	☐	Dual use research of concern
☒	☐	Plants

Methods

n/a	Involved in the study	
☒	☐	ChIP-seq
☐	☒	Flow cytometry
☒	☐	MRI-based neuroimaging

Antibodies

Antibodies used	anti-human CD24 FITC(ebioscience,11-0247-42), PE anti-human CD44(Biolegend, 338807), FITC anti-human CD45(Biolegend, 304005), FITC anti-human CD34(Biolegend, 343503), PE anti-human CD14(Biolegend, 367103), Anti-Hu CD19 PerCP-Cyanine5.5(eBioscience™, 45-0198-41), Anti-Human HLA-DQ APC(ebioscience, 17-9881-71).
Validation	the antibody was purchased from companies, please see above.

Eukaryotic cell lines

Policy information about cell lines and Sex and Gender in Research

Cell line source(s)	MDA-MB-231 cell line, purchased from ATCC , and cells derived from xenograft nude mice of human breast cancer MDA-MB-231 cells
Authentication	STR testing
Mycoplasma contamination	The cell line was test negative for Mycoplasma contamination
Commonly misidentified lines (See ICLAC register)	No commonly misidentified cell lines were used

Animals and other research organisms

Policy information about studies involving animals; ARRIVE guidelines recommended for reporting animal research, and Sex and Gender in Research

Laboratory animals	All mouse experiments were performed under the ethical guidelines of the University of Macau (animal protocol number: UMAEC-037-2015). Female nude mice with 6 weeks old were used for tumor inoculation in the work.
Wild animals	The study did not involve wild animals. Mice were housed in the SPF animal facility and monitored every alternate day for tumorigenesis. When the tumor was palpable, a caliper was used to measure the tumor size in two dimensions (length and width). Tumor volumes were calculated using the formula a × b2/2 (a and b represent the longest and shortest diameters, respectively) . When the tumor volume of the mice increased to 0.1–0.3 cm3, the mice were anesthetized using avertin (250 mg/kg), and a core biopsy needle (16G × 9 cm, 1 cm sample groove) was used to remove the primary tumor samples from the mice. For single drug screening part,the mice were divided into three groups, for two drugs and PBS treatment, respectively. For combinational drug screening research part, the mice were divided into four groups, for two individual drugs, the combinational drug and PBS treatment, respectively. Before each injection time, the mice were weighed, and tumor volume was measured and calculated. The treatment period was one month. Mouse was euthanasia with CO2 exposure after the experiment.
Reporting on sex	Male nude mice are more aggressive. To avoid the effect of the individual bold among the mice on the drug treatment results, we chose female nude mice. Sixty-five mice were used in the experiments and labeled with numbered ear tags. The tumor growth speed of each mouse differed due to mouse-to-mouse variations; the mice with tumors larger or smaller than the majority were discarded in the experiment to keep the tumor volume from the experimental nude mice almost the same before any drug treatment. A total of 21 mice were used for sample collection in the single drug screening research part; 20 mice were used for sample collection in the combinational drug screening research part.
Field-collected samples	During the study, the mice were monitored every alternate day. When the tumor was palpable, a caliper was used to measure the tumor size in two dimensions (length and width). Tumor volumes were calculated using the formula a × b2/2 (a and b represent the longest and shortest diameters, respectively) . When the tumor volume of the mice increased to 0.1–0.3 cm3, the mice were anesthetized using avertin (250 mg/kg), and a core biopsy needle (16G × 9 cm, 1 cm sample groove) was used to remove the primary tumor samples from the mice. For single drug screening part,the mice were divided into three groups, for two drugs and PBS treatment, respectively. For combinational drug screening research part, the mice were divided into four groups, for two individual drugs, the combinational drug and PBS treatment, respectively. The mice were divided into three groups, for two drugs and PBS treatment, respectively. Before each injection time, the mice were weighed, and tumor volume was measured and calculated. The treatment period was one month. The detailed methods can be found in the supporting information.
Ethics oversight	University of Macau

Note that full information on the approval of the study protocol must also be provided in the manuscript.

Clinical data

Policy information about clinical studies

All manuscripts should comply with the ICMJE guidelines for publication of clinical research and a completed CONSORT checklist must be included with all submissions.

Clinical trial registration	BSERE21-APP015-IME
Study protocol	The tumor was obtained from patients in the surgery room, which was supposed to be thrown away. In our experiment, the tumor

Study protocol	was dissociated into a cell suspension and added to the digital microfluidic chips to be co-cultured with target drugs (the detailed process is described in the method of supporting information).
Data collection	To successfully conduct the study, we collected two sets of data. The first set involved on-chip in-vitro drug screening of primary tumor cells using various drugs. Based on the screening results, we identified drugs that exhibited higher toxicity to the tumor cells and vice versa. The second set of data involved monitoring tumor recurrence in patients for six months after treatment with a drug selected by a doctor without any knowledge of the on-chip screening results. We collected patient data through follow-up investigations conducted by collaborating doctors from Liver Transplantation Center, The Third Affiliated Hospital, Sun Yat-Sen University, Guangzhou, China. The recruitment of patients took place between 8 June 2022 to 30 November 2022.
Outcomes	The viability of the cells on the chip served as the outcome measure for the effect of drugs during the screening process. On the other hand, the recurrence of the tumor in patients six months after surgery served as the outcome measure for the clinical treatment using the targeted drug. It is important to note that the doctors selecting the drug for the patients do not have access to the on-chip screening results.

Plants

Seed stocks	no plants in the work.
Novel plant genotypes	no novel plant genotypes in the work.
Authentication	no plants involved in the work.

Flow Cytometry

Plots

Confirm that:

☒ The axis labels state the marker and fluorochrome used (e.g. CD4-FITC).

☒ The axis scales are clearly visible. Include numbers along axes only for bottom left plot of group (a 'group' is an analysis of identical markers).

☒ All plots are contour plots with outliers or pseudocolor plots.

☐ A numerical value for number of cells or percentage (with statistics) is provided.

Methodology

Sample preparation	The sample of clinical liver patients were collected, digested with collagenase, and the cells were re-suspended to make a single-cell suspension, and counted. Then cells were washed with PBS twice (centrifugation at 800rpm for 5min), and cell concentration was adjusted to 1×107/ml cells by medium. 100 uL of cells for each marker. 2μL of antibody (1:50) were added and mix well, including anti-human CD24 FITC(ebioscience,11-0247-42), PE anti-human CD44(Biolegend, 338807), FITC anti-human CD45(Biolegend, 304005), FITC anti-human CD34(Biolegend, 343503), PE anti-human CD14(Biolegend, 367103), Anti-Hu CD19 PerCP-Cyanine5.5(eBioscience™, 45-0198-41), Anti-Human HLA-DQ APC(ebioscience, 17-9881-71). Reaction at room temperature, away from light for 30min. The cells were washed twice with PBS (centrifugation at 800rpm for 5min).
Instrument	Flow Cytometer (FACSDiva, Version 6.1).
Software	Flow cytometry was performed for observation and detection by instrument Flow Cytometer (FACSDiva, Version 6.1).
Cell population abundance	In the gated cell population cells were run to achieve > 10,000 events.
Gating strategy	Gating was performed based on identifying a distinct population in FSC vs SSC plots.

☐ Tick this box to confirm that a figure exemplifying the gating strategy is provided in the Supplementary Information.
